# High-resolution mapping of tuberculosis transmission: Whole genome sequencing and phylogenetic modelling of a cohort from Valencia Region, Spain

**DOI:** 10.1371/journal.pmed.1002961

**Published:** 2019-10-31

**Authors:** Yuanwei Xu, Irving Cancino-Muñoz, Manuela Torres-Puente, Luis M. Villamayor, Rafael Borrás, María Borrás-Máñez, Montserrat Bosque, Juan J. Camarena, Ester Colomer-Roig, Javier Colomina, Isabel Escribano, Oscar Esparcia-Rodríguez, Ana Gil-Brusola, Concepción Gimeno, Adelina Gimeno-Gascón, Bárbara Gomila-Sard, Damiana González-Granda, Nieves Gonzalo-Jiménez, María Remedio Guna-Serrano, José Luis López-Hontangas, Coral Martín-González, Rosario Moreno-Muñoz, David Navarro, María Navarro, Nieves Orta, Elvira Pérez, Josep Prat, Juan Carlos Rodríguez, María Montserrat Ruiz-García, Herme Vanaclocha, Caroline Colijn, Iñaki Comas

**Affiliations:** 1 Centre for Mathematics of Precision Healthcare, Department of Mathematics, Imperial College London, London, United Kingdom; 2 Instituto de Biomedicina de Valencia, Consejo Superior de Investigaciones Científicas, Valencia, Spain; 3 Genomics and Health Unit, FISABIO Public Health, Valencia, Spain; 4 Microbiology Service, Hospital Clínico Universitario, Valencia, Spain; 5 Microbiology and Parasitology Service, Hospital Universitario de La Ribera, Alzira, Spain; 6 Microbiology Service, Hospital Arnau de Vilanova, Valencia, Spain; 7 Microbiology Service, Hospital Universitario Dr. Peset, Valencia, Spain; 8 Microbiology Laboratory, Hospital Virgen de los Lírios, Alcoy, Spain; 9 Microbiology Service, Hospital de Denia, Denia, Spain; 10 Microbiology Service, Hospital Universitari i Politècnic La Fe, Valencia, Spain; 11 Microbiology Service, Hospital General Universitario de Valencia, Valencia, Spain; 12 Microbiology Service, Hospital General Universitario de Alicante, Alicante, Spain; 13 Microbiology Service, Hospital General Universitario de Castellón, Castellon, Spain; 14 Microbiology Service, Hospital Lluís Alcanyis, Xativa, Spain; 15 Microbiology Service, Hospital General Universitario de Elche, Elche, Spain; 16 Microbiology Service, Hospital Universitario de San Juan de Alicante, Alicante, Spain; 17 Microbiology Service, Hospital de la Vega Baixa, Orihuela, Spain; 18 Microbiology Service, Hospital San Francesc de Borja, Gandía, Spain; 19 Subdirección General de Epidemiología y Vigilancia de la Salud, Dirección General de Salud Pública, Valencia, Spain; 20 Microbiology Service, Hospital de Sagunto, Sagunto, Spain; 21 Department of Mathematics, Simon Fraser University, Burnaby, British Columbia, Canada; Harvard Medical School, UNITED STATES

## Abstract

**Background:**

Whole genome sequencing provides better delineation of transmission clusters in *Mycobacterium tuberculosis* than traditional methods. However, its ability to reveal individual transmission links within clusters is limited. Here, we used a 2-step approach based on Bayesian transmission reconstruction to (1) identify likely index and missing cases, (2) determine risk factors associated with transmitters, and (3) estimate when transmission happened.

**Methods and findings:**

We developed our transmission reconstruction method using genomic and epidemiological data from a population-based study from Valencia Region, Spain. Tuberculosis (TB) incidence during the study period was 8.4 cases per 100,000 people. While the study is ongoing, the sampling frame for this work includes notified TB cases between 1 January 2014 and 31 December 2016. We identified a total of 21 transmission clusters that fulfilled the criteria for analysis. These contained a total of 117 individuals diagnosed with active TB (109 with epidemiological data). Demographic characteristics of the study population were as follows: 80/109 (73%) individuals were Spanish-born, 76/109 (70%) individuals were men, and the mean age was 42.51 years (SD 18.46). We found that 66/109 (61%) TB patients were sputum positive at diagnosis, and 10/109 (9%) were HIV positive. We used the data to reveal individual transmission links, and to identify index cases, missing cases, likely transmitters, and associated transmission risk factors. Our Bayesian inference approach suggests that at least 60% of index cases are likely misidentified by local public health. Our data also suggest that factors associated with likely transmitters are different to those of simply being in a transmission cluster, highlighting the importance of differentiating between these 2 phenomena. Our data suggest that type 2 diabetes mellitus is a risk factor associated with being a transmitter (odds ratio 0.19 [95% CI 0.02–1.10], *p* < 0.003). Finally, we used the most likely timing for transmission events to study when TB transmission occurred; we identified that 5/14 (35.7%) cases likely transmitted TB well before symptom onset, and these were largely sputum negative at diagnosis. Limited within-cluster diversity does not allow us to extrapolate our findings to the whole TB population in Valencia Region.

**Conclusions:**

In this study, we found that index cases are often misidentified, with downstream consequences for epidemiological investigations because likely transmitters can be missed. Our findings regarding inferred transmission timing suggest that TB transmission can occur before patient symptom onset, suggesting also that TB transmits during sub-clinical disease. This result has direct implications for diagnosing TB and reducing transmission. Overall, we show that a transition to individual-based genomic epidemiology will likely close some of the knowledge gaps in TB transmission and may redirect efforts towards cost-effective contact investigations for improved TB control.

## Author summary

**Why was this study done?:**

- To facilitate public health intervention and to design new tuberculosis (TB) control strategies, there is a need to identify when TB is transmitted and by whom.
- Whole genome sequencing combined with phylogenetic modelling has the potential to fill knowledge gaps on TB epidemiology.

**What did the researchers do and find?:**

- We analyzed a population-based cohort of TB patients in Valencia Region, Spain, between 2014 and 2016.
- We systematically sequenced the whole genomes of culture positive isolates and identified transmission clusters. We combined genomic and epidemiological data to understand how TB is transmitted.
- We showed that in many cases the index case is likely either not sampled or not the first diagnosed.
- For a fraction of TB individuals, we could accurately predict when transmission happened. For several transmitters we showed that transmission happened well before diagnosis and symptom onset.

**What do these findings mean?:**

- Our findings provide novel insights into TB transmission going beyond cluster delineation.
- Our results highlight the limitation of contact tracing to identify index cases and can be used to design new TB control strategies.
- The finding that TB can be transmitted by individuals before they have symptoms, very likely during sub-clinical or incipient disease, has important implications for TB control strategies. More studies are needed to understand the dynamics of TB transmission in different clinical settings.
- This study highlights the importance of combining genomic data with epidemiological data, in order to gain new insight into how and when TB is transmitted.

## Introduction

Better understanding of tuberculosis (TB) transmission is key for TB control in the 21st century. Economic resources are very limited in many high-burden countries, while in low-burden countries, TB control is jeopardized by diminishing resources, as TB is not perceived as a public health issue [1]. The limited funding is spent on tracing contacts of individuals diagnosed with TB; many of these contacts test negative for TB infection, whereas other contacts that had substantial exposure may not be screened. Historically a dichotomy between active and latent disease has been used at the epidemiological level to differentiate those TB cases that can transmit (active TB disease) versus those that do not (latent). However, more recent evidence suggests that the transition between these different states is fuzzy, and that TB development may be better represented as a spectrum of clinical and sub-clinical states [2]. The degree to which sub-clinical disease contributes to transmission is largely unknown, particularly because tools to detect sub-clinical disease have only recently become available [3,4].

Whole genome sequencing (WGS) of patient isolates shows a higher agreement with contact investigations than previous markers [5]. Importantly WGS is also a superior tool to delineate transmission clusters and can be used to estimate the burden of transmission [6]. But only very limited approaches have been developed using WGS to identify individual transmission links. Phylodynamic and transmission network analyses based on the combined use of WGS and epidemiological data have been primarily confined to the analysis of large outbreaks [7–10]. However, transmission clusters spanning decades are more an exception than a rule in TB epidemiology [11,12]. For most epidemiological scenarios, 2 key limitations prevent the use of phylodynamic and network models to predict transmission links: the diversity of the bacteria is extremely low, and the time span does not allow a good correlation between time and the accumulation of variation.

Population-based analyses where dozens or hundreds of transmission clusters can be identified typically involve cluster sizes of 1–15 TB cases and sampling times of 2–5 years. In high-burden countries, cluster sizes may be larger but time frames are still short. We thus developed an approach that allowed us to simultaneously analyze small clusters from a 3-year population-based study in the Valencia Region of Spain. Our approach infers index cases as well as estimating transmission times.

## Methods

Our overall analysis proceeded as follows: isolate collection, sequencing analysis, identification of transmission clusters meeting certain criteria, phylogenetic tree reconstruction, calculation of tree timing with several choices of molecular clock rate, and, finally, Bayesian transmission analysis.

### Case definitions

#### Clustered case

A clustered case is a case that is genomically close to another case in the population according to a genetic threshold. Typically, for recent transmission, 12 or 5 SNPs are used but see below.

#### Index case

The index case is the first documented individual in a TB outbreak, usually the one that generates an epidemiological investigation. In most epidemiological investigations in TB, this coincides with (or it is assumed to be) the first diagnosed individual.

#### Most likely ancestral genotype (MLAG)

The MLAG is the reconstructed genotype of a hypothetical ancestral case of an outbreak. It may coincide or not with the index case from the epidemiological investigation. A match of the MLAG with any sampled genotype suggests that the sampled genotype is likely an index case.

### Ethics statement

This study was approved by the Ethics Committee for Clinical Research of the Valencia Regional Public Health Agency (Comité Ético de Investigación Clínica de la Dirección General de Salud Pública y Centro Superior de Investigación en Salud Pública). Informed consent was waived on the basis that TB is part of the regional compulsory surveillance program of communicable diseases. All personal information was anonymized, and no data allowing individual identification was retained.

### Study population and isolate collection

Valencia Region has 4,974,475 million inhabitants and is composed of 3 provinces, Castellón, Valencia, and Alicante. In 2018, there were 315 reported individuals with TB in the entire region (incidence rate of 6.4/100,000 inhabitants); Valencia is considered a low-TB-burden region. Contact tracing investigation is the gold standard procedure to detect transmission clusters and is done in 74.1% of all notified TB cases.

We performed a population-based genomic study involving 785 TB culture positive cases in Valencia Region, Spain, during 2014–2016 as a part of an ongoing local genomic epidemiology study. Using WGS data to delineate transmission (based on SNP distances, cutoff of ≤15 SNPs; see below), we identified 121 clusters, most of which involved 2 cases per cluster (*n* = 325 clustered cases; see S1 Text). For the present analysis we included all transmission clusters that involved at least 4 TB cases and had more than 1 SNP (variant) between the strains. Based on a reviewer’s feedback, we performed a chi-squared test to corroborate that the clusters selected for this study were a good representation of the total number of clustered cases in the population.

A total of 21 clusters met the criteria, involving a total 117 people with TB. For 115 of these we had epidemiological data including date of diagnosis and diagnostic symptom onset as well as other clinical and demographic data. For 2 individuals we used the date of culture positivity with a 2-week correction to infer the date of diagnosis.

### WGS analysis and transmission delineation

DNA from TB culture positive Mycobacteria Growth Indicator Tubes (Becton Dickinson) was extracted. Sequencing libraries were constructed with Nextera XT DNA Library Prep Kit (Illumina) and sequenced on the Illumina MiSeq instrument. Generated paired-end sequencing reads were trimmed, and likely contaminant reads that might be present in clinical culture were filtered using KRAKEN software [13]. The bioinformatic analysis was performed following a previous pipeline [14]. Briefly, sequencing reads were mapped and aligned to an inferred *Mycobacterium tuberculosis* complex (MTBC) most likely common ancestor genome. Next, variants were separated into INDELS (small insertions and deletions) and SNPs. Variants with at least 10 reads in both strains and a quality score of 20 were selected. Because we wanted to detect genomic transmission, we focused on SNPs that were present with at least a 90% frequency. Finally, SNPs annotated in regions difficult to map such as repetitive sequences and PPE/PE-PGRS genes were removed from the analysis, as well as those detected in a window of 10 variants near INDELS. In addition, variants known to confer drug resistance [15,16] were removed.

This pipeline has been validated by international public health TB reference laboratories (http://tgu.ibv.csic.es/?page_id=1794) and published [17,18]. The parameters used in the pipeline are common among the genomic TB research community [19].

### Transmission cluster delineation based on SNP distances and phylogeny

Transmission clusters were defined using a loose cutoff of ≤15 SNPs. Furthermore, all detected groups were confirmed by building a phylogeny that included all the isolates. This phylogeny was inferred using the maximum likelihood phylogenetic approach with RAxML v8.2 [20], applying the General Time Reversible model of nucleotide substitution with the gamma distribution (GTRGAMMA). Transmission clusters with more than 1 SNP between the strains and composed of at least 4 TB individuals were kept for ensuing analyses. The methods described below are agnostic to the cutoff value, but with a threshold of 15 SNPs, we were sure to incorporate recent and old transmission events. In any case, most samples were below the cutoff of 12 SNPs, and 82% were below the cutoff of 5 variants.

### Reconstruction of genetic relatedness networks

The resulting SNP alignment for each cluster was used to infer a genetic relatedness network. Due to the monomorphic and non-recombining nature of the MTBC [21] and the possibility that the ancestral genotype was present in the samples, we used a parsimony-based algorithm for network reconstruction implemented in the PopART software [22]. We chose a median joining network (MJN) approach because it allows cases to occupy central positions in the network; genotypes at branching points in the parsimony tree are hypothesized to have been present but unsampled. In addition, a reconstructed recent ancestor of the cluster based on the phylogenetic topology was added to the network so we could (1) hypothesize the MLAG and (2) infer the directionality of a SNP (wild-type versus mutant status) given the MLAG. In the genetic network analysis, we considered that any strain matching the MLAG for its transmission cluster was a candidate to be the index case of the cluster.

### Timed tree reconstruction

The accepted value for the substitution rate in TB is approximately 0.3–0.5 substitutions per genome per year [8,23], though our data seem to suggest that this rate may vary both between clusters and at the individual lineage level within clusters. We first estimated timed trees for all clusters using the treedater package in R [24] with 5 different clock rate values (ranging from 0.327 to 1.103) sampled from a log-normal distribution following a meta-analysis. Although we generated predictions for a range of rates, for clarity, results in the main text will be based on a clock rate of 0.363, which closely matches the mean rate identified in our meta-analysis and in a recent publication [25] for MTBC lineage 4, which dominates our population. Parameters used to obtain the different clock rate values, as well as the meta-analysis performed, are described and shown in S1 Text and S1 Table.

### Transmission inference

We developed a method of simultaneous transmission inference on many clusters based on TransPhylo, a Bayesian analysis approach that uses the Markov chain Monte Carlo (MCMC) method to reconstruct transmission trees from pathogen phylogeny [26]. The main difference between our method and TransPhylo’s previous capabilities is that we can perform inference with multiple transmission clusters simultaneously, choosing which parameters should be shared between clusters.

The resulting transmission tree contains information about who infected whom and when, and also whether a case is sampled or not. This information is represented by a matrix whose columns are the times of infection, times of sampling, and transmitters, and whose rows correspond to individuals in the cluster. If an individual in the reconstructed tree is not sampled, then the corresponding entry for time of sampling is empty. TransPhylo produces a posterior sample of such trees. From this collection, we can extract (1) the posterior probability that the index case of a cluster is sampled and (2) the posterior probability that each host transmitted TB in their cluster. A detailed protocol that includes all equations of the TransPhylo method can be found in S1 Text.

In order to test and validate our method, we performed simulations of 2 outbreaks. We observed narrower widths of credible intervals for all parameters (S2 Text and S1 and S2 Figs) using the simultaneous approach. This method has been incorporated into the latest version of the TransPhylo package [26].

### Statistical analysis

We selected the index cases and the samples with higher than 0.6 posterior probability of being transmitters as predicted by TransPhylo (23 transmitters compared to the remaining 84 clustered cases), with sensitivity analysis of the later threshold in S1 Text and S1 Table. Then, we computed the odds ratio (OR) and 95% confidence intervals (Fisher’s exact test) to explore epidemiological variables associated with being a transmitter. Furthermore, we performed a multivariate logistic regression to confirm our univariate result. Based on peer review feedback, we statistically compared epidemiological variables associated with transmitters to those of the non-clustered cases identified in the whole dataset.

## Results

### Genetic networks suggest missing index cases

Using an initial threshold of 15 SNPs, we identified a total of 21 transmission clusters involving 117 TB cases (Table 1). This 15-SNP threshold allowed us to look at older transmission events, although most of the cases (81.2%) were within 5 SNPs of another case, consistent with very recent transmission. Most of the clusters had more than 1 case with an identical genotype (0 SNP difference); 5 clusters had no identical pairs (S2 Table). No statistical difference was observed for available clinical, epidemiological, and demographic variables between the 21 transmission clusters that met our inclusion criteria (*n* = 109) and the total clustered samples in the population (*n* = 325) (see S1 Text and S3 Table).

**Table 1:** Main characteristics of the study population.

Characteristic	All patients (*n* = 109)*
**Age (years)**	
<18	11 (10%)
19–34	20 (18%)
35–65	66 (61%)
>65	12 (11%)
**Sex**	
Female	33 (30%)
Male	76 (70%)
**Place of birth**	
Spain	80 (73%)
Other country	29 (27%)
**Sputum smear**	
Positive	66 (61%)
Negative	41 (38%)
**Disease type**	
Pulmonary	100 (92%)
Extrapulmonary	9 (8%)
**Alcoholism**	25 (23%)
**Diabetes**	13 (12%)
**HIV infected**	10 (9%)
**Social exclusion**	13 (12%)
**Healthcare worker**	5 (5%)
**Imprisonment**	8 (7%)
**Diagnostic delay (days)**	
≤30	46 (42%)
31–60	25 (23%)
61–89	14 (13%)
≥90	32 (29%)

*Eight TB cases had no epidemiological data.

Genetic networks are a popular approach to try to understand transmission without the need for additional epidemiological data. Using the SNP alignment data, we applied the MJN algorithm to establish genetic relatedness between the strains. A total of 22 missing links were predicted (involving 14 out of 21 genetic networks). In 5 of the genetic networks the predicted missing genotype corresponded to the MLAG, suggesting that the index case was not sampled. In other clusters intermediate genotypes were missing. In contrast, in 7 networks (33%) we did not predict any missing links, indicating that the MLAG predicted was present among the TB cases analyzed.

**Fig 1:**
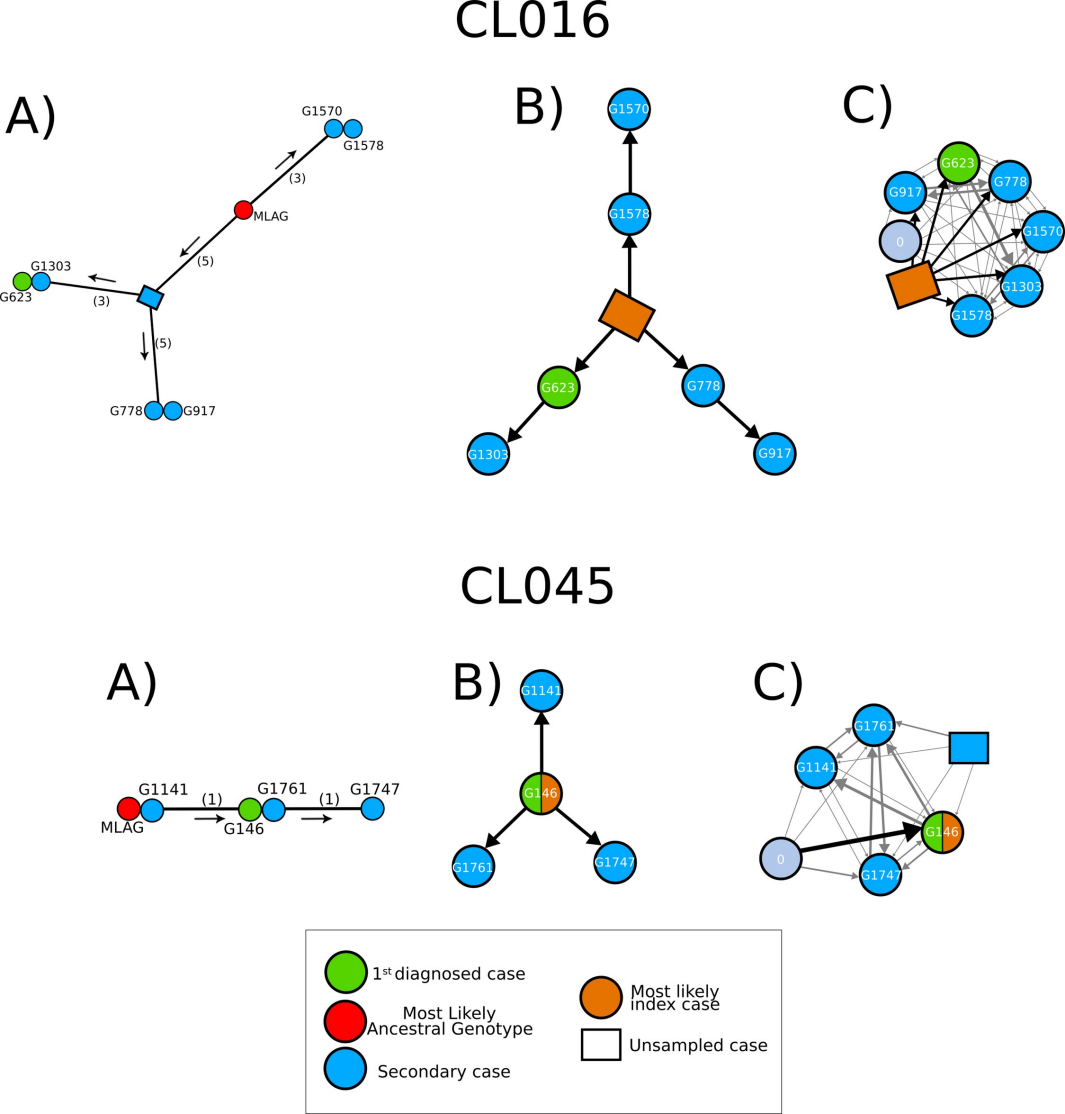
Comparison of transmission reconstruction methods. The figure shows for clusters CL045 and CL016 the inferred genetic network (A) and the consensus transmission tree inferred from TransPhylo (B and C). In addition we show the strength of the TransPhylo prediction (C). When the index case is sampled, it is depicted by a direct black arrow connecting the grey “0” circle to the respective individual. This is the case for G146 in CL045. When the index case is missing, this is represented by an orange square connected to all cases, as in CL016. Any other unsampled tuberculosis case is shown using a blue square symbol.

In the MJN approach it is reasonable to estimate that the strain with the same genotype as the MLAG is also the most likely index case. However, in several clusters (Figs 1, S3 and S4), more than one strain matched the MLAG, and thus the approach, which is based solely in genotypes, cannot predict which of the matching cases is the most likely index case. One striking feature of the networks in which we can identify an MLAG among sampled TB cases is that this hypothetical index case does not always coincide with the first diagnosed case (Fig 1A). This situation occurred in 2 of the 5 networks in which there was a case with the same genotype as the MLAG (clusters CL045 and CL078). Together with the fact that in an additional 14 genetic networks the MLAG was not present, this suggests that the common assumption that the earliest diagnosed case is the index case is not necessarily correct. All the networks reconstructed by the genetic network approach can be found in S3 and S4 Figs.

Genetic networks do not necessarily reflect transmission, as they do not integrate key information. For instance, the number of substitutions observed is affected by the time elapsed since infection and by within-host diversity; multiple clones can coexist in the same individual, and they may be differentially transmitted. Thus, the assumption that the SNPs are gained from an ancestral reconstructed genotype and that diversification events represent transmission events may not be correct.

### TransPhylo identifies index cases not detected by contact tracing

**Fig 2:**
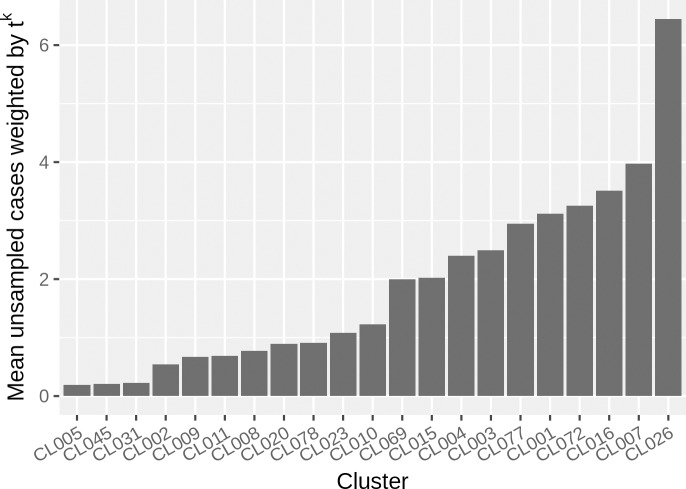
Weighted mean number of unsampled tuberculosis cases. For each posterior transmission tree, we associate a weighting factor *t*^*k*^, where *k* is the number of sampled cases for which transmission happened after diagnosis, and *t* = 0.1. This accounts for the fact that individuals are treated once diagnosed, and so are less likely to transmit. This figure shows the mean number of unsampled cases for one of the simulated clock rates (0.363). The results for all clock rates appear in S5 Fig.

The TransPhylo approach integrates sample timing and genetic relatedness, and allows for within-host diversity, thereby avoiding the assumption that diversification represents transmission. TransPhylo produces posterior reconstructed transmission events and timing for each cluster, which can be visualized in many ways, including consensus trees (Fig 1B) and the posterior probability of infection between cases (Fig 1C). In our study, TransPhylo estimated that there were unsampled cases, with different numbers of unsampled cases in different clusters. For the main results, we selected a clock rate value of 0.363 SNPs/genome/year, which is the rate obtained by others [23,27]. The results show that most transmission clusters had 2 or fewer unsampled cases (62%). Only 1 cluster (CL026) had a median number of unsampled cases greater than 5 (Fig 2). The estimated number of unsampled cases is lower if a higher substitution rate is assumed, with very few unsampled cases under a fast clock assumption (S5 Fig). This effect occurs because with a faster assumed clock rate, timed tree branches are shorter, and TransPhylo is less likely to place unsampled cases along the branches.

**Fig 3:**
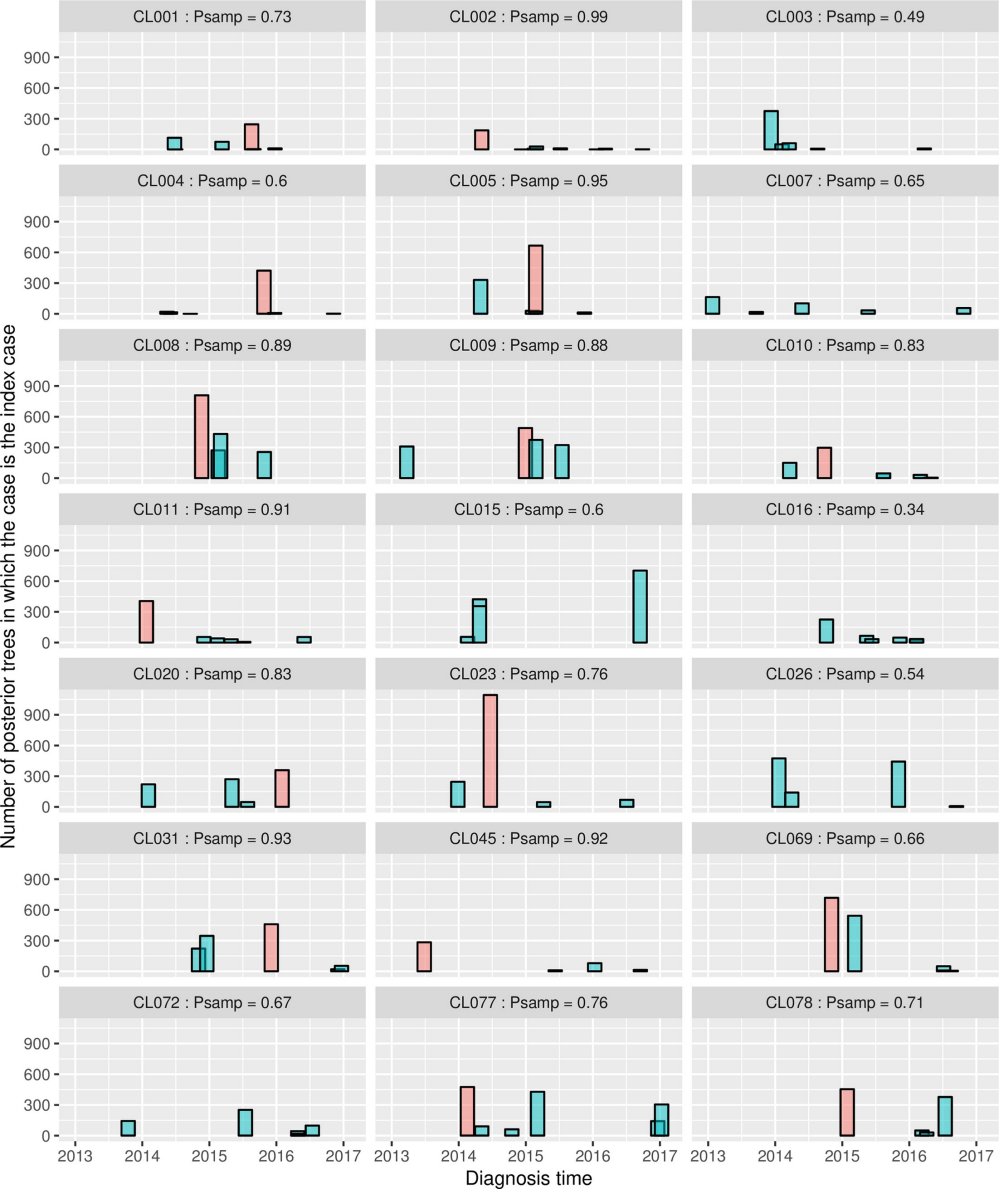
The posterior probability that each individual is the index case for a cluster versus the time of diagnosis of the individual. The individual with highest posterior probability to be the index case is shown in red for each cluster. In some clusters, the first diagnosed case was the estimated index case, in that it had the highest probability of being the index case (e.g., CL002). In contrast, in the majority of clusters the most likely index case was not the first diagnosed individual (e.g., CL010 and CL023) or was not sampled (e.g., CL016 and CL003). The *P*samp values are the posterior probability that the index case was *any* of the sampled individuals—in some clusters (e.g., CL003) the index case was likely to have been an unsampled individual.

TransPhylo’s augmented MCMC approach allows us to extract the inferred index case for each posterior tree. Fig 3 shows for every cluster the probability that each diagnosed individual in the cluster was the index case, along with the individuals’ diagnosis times. There are 6 clusters in which the index case was most likely unsampled. For those clusters where the index case was likely sampled, the index case is not always the first diagnosed individual (33%); the index case’s diagnosis can be many months after the first diagnosis (e.g., CL005). Most of the clustered cases were not detected as contacts in the contact tracing epidemiological investigations.

There is general agreement between TransPhylo and the genetic network approach in identifying those clusters in which the index case is likely sampled. For 7 clusters (33%), both approaches predicted that the index case had been sampled. TransPhylo predicted the presence of an index case in 8 additional clusters in which the exact MLAG genotype did not occur, and consequently the genetic network approach did not predict that the index case was sampled. For the rest of the clusters (*n* = 6), neither TransPhylo nor the genetic network identified a likely index case. However, despite this general agreement, the methods do not always agree on which patient was the likely index case.

Genetic networks predicted the same index case as TransPhylo in only 2 (13%) of the 15 clusters with a likely sampled index case. This disagreement is likely associated with the fact that the time of sampling and rate of genetic change are not taken into account in the genetic network prediction. Also, the genetic network approach predicted more unsampled genotypes than TransPhylo, reflecting the fact that some of the missing genotypes likely existed but evolved within a host and were not transmitted (S3 and S4 Figs).

### Timing of events reveals TB cases transmitting before diagnosis or symptom onset

Because it integrates information about case timing and the molecular clock alongside genetic relatedness of isolates, TransPhylo can estimate the timing of transmission, which can be compared to diagnosis times and reported symptom times. Thus, triangulation of relevant dates and timing should allow us to use TransPhylo to evaluate how much transmission could be averted by earlier identification of individuals with TB or by isolating patients during the first stages of treatment.

**Fig 4:**
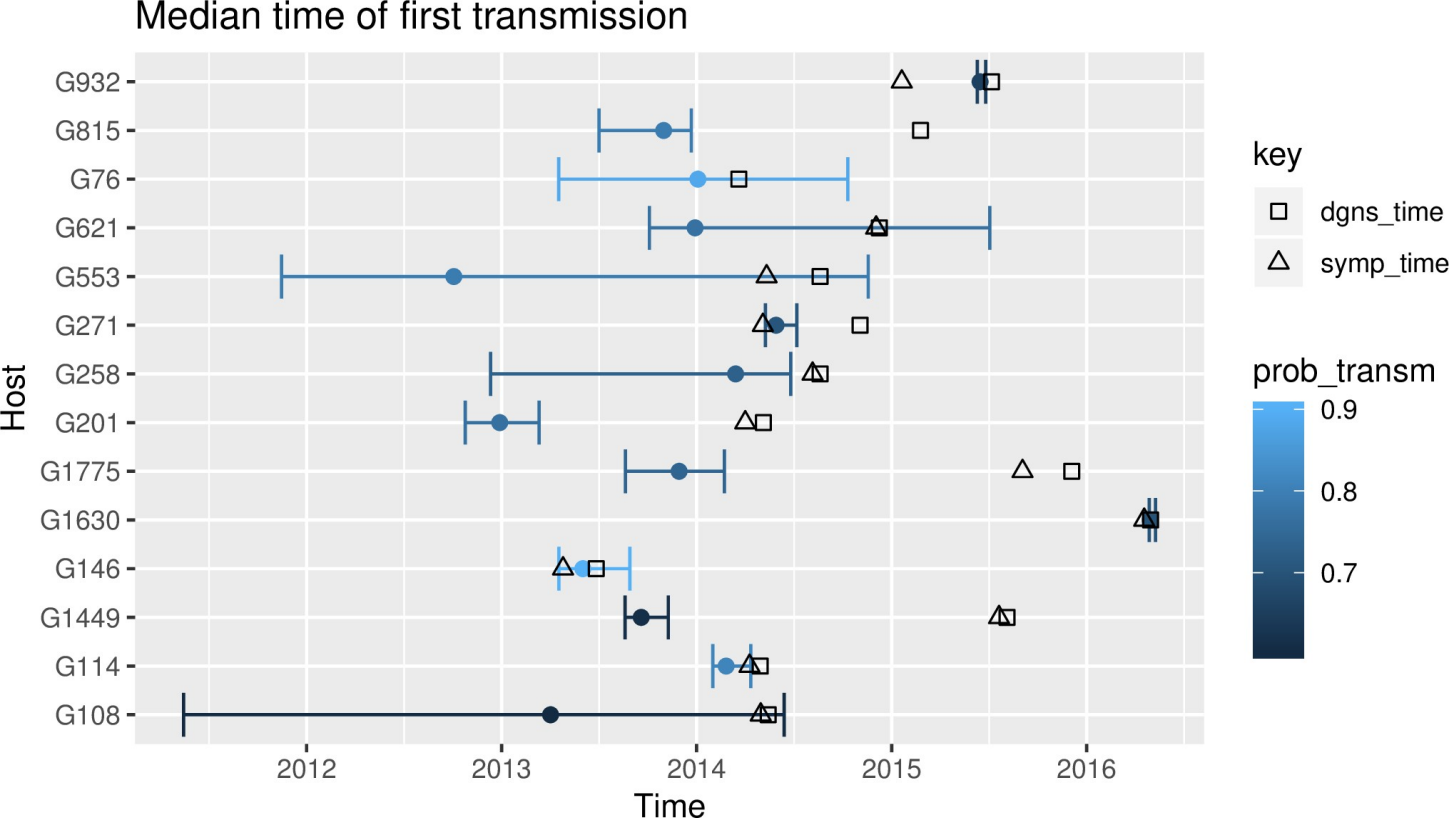
Resampled median time of first transmission. The graph represents the median time of the first highly likely transmission for individuals for whom the posterior probability of transmitting (prob_transm) was greater than 0.6, under a clock rate value of 0.363 SNPs/genome/year. For each case, the diagnosis time (dgns_time; squares) and, where known, the symptom onset time (symp_time; triangles) are added. Analogous graphs for different transmission probability cutoffs, and without cutoffs, are shown in S6–S12 Figs.

First, we extracted transmission trees corresponding to one of the molecular clock rates (0.363 SNPs/genome/year) and selected all individuals for whom the probability of transmitting was greater than 0.6. We then compared inferred transmission times to diagnosis times and to the reported times of symptom onset. A total of 14 individuals had a high likelihood of being transmitters (Fig 4). We reasoned that if our prediction was accurate, many transmission events should happen between the onset of symptoms and diagnosis; this is the case for 9 out of the 14 TB individuals. However, when we looked at the time of transmission in the other 5 cases, transmission occurred before symptom onset or diagnosis (G815, G258, G201, G1775, and G1449). Notably, 3 out of the 5 individuals were sputum negative at the time of diagnosis, suggesting that they were infectious before, but not at the time of detection. The time of first transmission event for all cases in every cluster is reported in S6–S12 Figs, including combinations of different probabilities and clock rates.

To evaluate the feasibility that transmission happened before symptoms, we analyzed the contact tracing and epidemiological data available for 1 of the cases. G1449 was a credible transmitter before symptom onset (Fig 4). G1449 clustered with another case, G1011, which was the 18-year-old daughter of G1449. Both were identified almost simultaneously, but the daughter was the first to seek care. Thus, she was considered the index case, and contacts were screened. G1449 was identified during screening a few days later. We estimate that G1449 infected G1011 less than 2 years before, which is compatible with the incubation time of latent TB in persons without known risk factors. Conversely, if G1011 infected G1449 after symptom onset, then G1449 had to develop symptoms in less than 1 month since infection, which is less likely than the other scenario.

We also reasoned that the probability of transmission should be compatible with the known epidemiological characteristics of the patients. We used the time of arrival of foreign nationals to evaluate the feasibility that transmission happened when we predicted. In all individuals with a high probability of transmitting TB, transmission happened after arrival to the country. Conversely, there were 5 individuals for whom transmission was predicted to have happened before arrival, so for these individuals there is a contradiction between the prediction (if they were transmitters) and the epidemiological history. In all 5 cases, our approach did not identify them as credible transmitters (probabilities of transmission < 0.3; S4 Table).

Finally, we examined whether individuals with longer estimated times between infection and diagnosis had higher numbers of secondary TB cases. This would be expected, since delayed diagnosis gives an individual the opportunity to expose others and to become the index case of a cluster. We found that the estimated time to diagnosis was longer for those individuals predicted to have infected 2 or more secondary cases, but the results are variable, as expected given that many other factors affect probabilities of transmission and infection (see S13 Fig).

### Identification of transmitters allows association of risk factors to transmission

**Fig 5:**
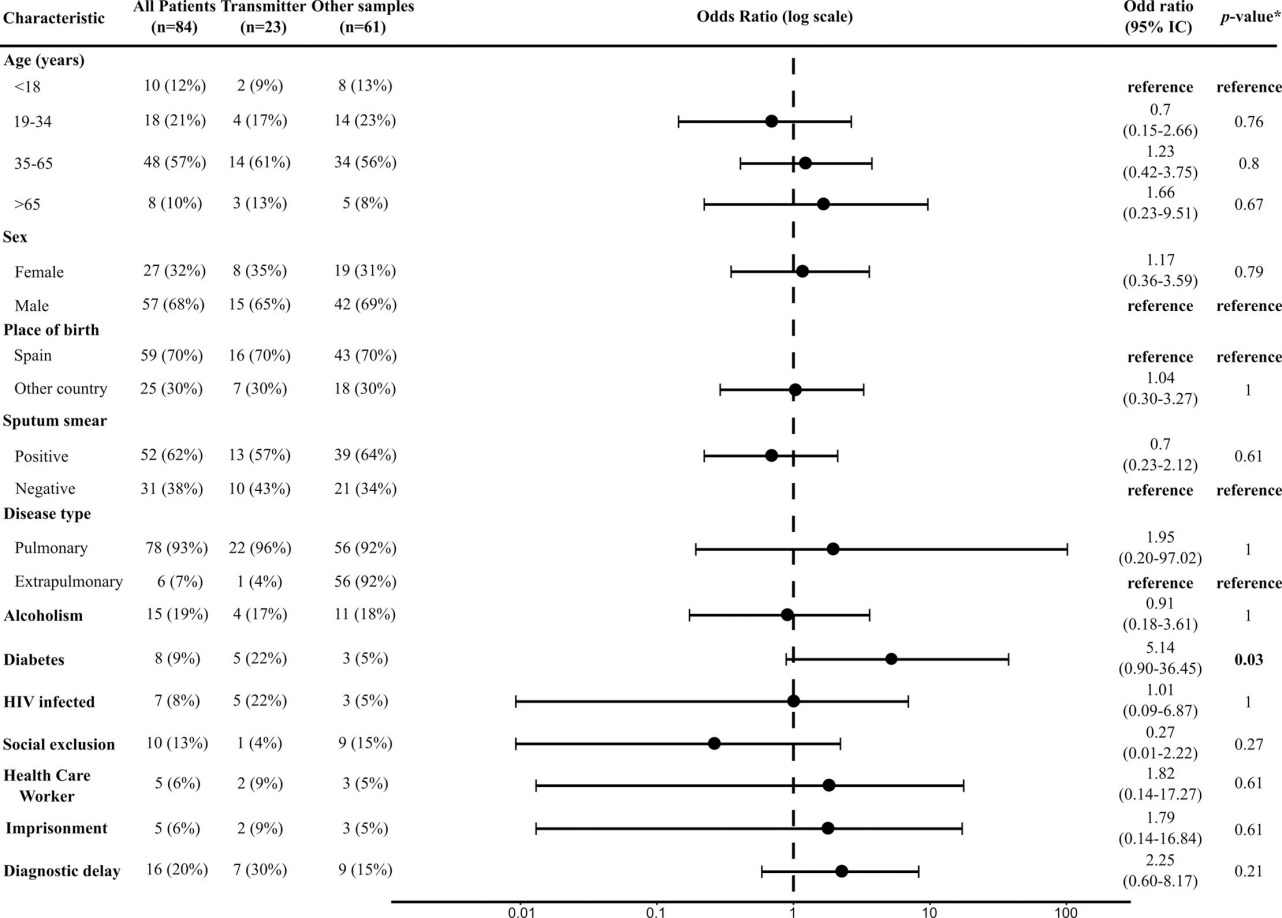
Epidemiological characteristics of the cases used to identify transmission risk factors. Note that the data do not include all the study samples: for 5 clusters we were not able to identify a likely transmission event, and these clusters were excluded from this analysis. Transmitters are defined as those individuals estimated to be likely transmitters and/or likely index cases detected by TransPhylo. The figure shows estimated odd ratios for each risk factor tested. *Fisher’s exact test. Comparisons were made between transmitter cases and the rest of the clustered samples.

For 66% of the clusters analyzed, the index case identified by TransPhylo was either unsampled or not the first diagnosed case (14 out of 21). This suggests that index cases based on diagnostic dates can be misleading. In addition, analyses of risk factors associated with transmission using molecular epidemiology data have been traditionally performed on group measures of clustering (clustered versus unique cases, association with cluster sizes). This approach obviates the fact that not all individuals with TB are transmitters, and thus risk factors associated with transmission are difficult to disentangle from those associated with infection. Our identification of likely index cases and transmitters allows us to explore whether risk factors have a different distribution specifically among likely transmitters. We combined likely transmitter cases together with the index cases predicted by TransPhylo (*n* = 23) and compared them to the other clustered cases (*n* = 61). Our statistical analysis is limited by the low number of clusters and the low number of transmitters that were unequivocally identified. Also, clustered cases are a composite of transmitters, non-transmitters, and those cases that cannot be confidently assigned to either category. Still, relevant differences between likely transmitters and the rest of the clustered cases can be identified (Fig 5).

As a proof of concept, transmitters tended to be diagnosed later (mean diagnostic delay 85 days versus 54 days), although this difference is not statistically significant. Other variables also suggest important differences between being a transmitter and simply being part of a cluster. Transmitters were significantly enriched in diabetic patients in both univariate (Fisher’s exact test; OR 0.19 [95% CI 0.02–1.10], *p* < 0.003) and multivariate (logistic regression; OR 23.77 [95% CI 2.53–339.69], *p* < 0.009) statistical analyses. It has been suggested before that diabetic patients tend to have larger TB cavities, a factor known to be associated with transmission [28]. Finally, we confirm previous reports showing that individuals who are smear negative at the time of diagnosis can be transmitters (37% in our dataset). However, we take these results with caution. We repeated the analysis comparing transmitters to non-clustered cases, and diabetes was still enriched (27% versus 10%), but not significantly (*p* = 0.06). While small sample sizes do not allow us to draw more conclusions, these preliminary results show the importance of differentiating between being a transmitter and being infected.

## Discussion

We present a genomic-based approach to unveil individual TB transmission links between patients within transmission clusters. Importantly, our method allows us to identify, or infer the absence of, the most likely index case, as well as estimate the number of unsampled cases within a cluster. These findings may contribute to reorienting contact investigation strategies in terms of to whom and where TB testing should be done. In addition, we identify potential transmission events during the sub-clinical disease stage, suggesting the need to incorporate early disease stages in epidemiological models and TB control programs.

WGS has been shown to be superior to previous genotyping tools in identifying TB cases likely to be of recent transmission [29]. Nevertheless, there is only an agreement of 30%–50% between those identified by WGS as TB cases of recent transmission and those identified by contact tracing [30]. This scenario indicates that likely index cases are missing, and improved contact investigation strategies are required in order to detect those individuals. A recent clinical trial [31] showed that close contacts of index cases identified by active case finding have better TB cure rates than those identified by passive case finding. Thus, identification of index cases has implications at the population and at the individual care level. In this study, we showed that in up to 28% of clusters there is no evidence that the index case is included among the individuals in the cluster. For those clusters in which an index case was detected, 60% of the time the index case was not the individual first diagnosed with TB, suggesting that efforts to identify transmission are imperfect.

The reasons that index cases are not sampled in a study may be multiple and will probably vary by clinical setting. First, index case transmission could have occurred prior to the sampling time. This is very likely in our analyses, where we potentially include older transmission events, though fixed SNP cutoffs may not perfectly delineate transmission clusters [32]. Furthermore, we missed those individuals with culture negative status at the time of diagnosis, and they may have contributed to transmission. However, it is worrying that individuals with TB may have been missed by control programs and may remain actively transmitting in the population. In Valencia, around 3,000 contacts are investigated every year following the European Centre for Disease Prevention and Control guidelines. Still, a large percentage of the clustered cases were not identified as contacts, consistent with similar published studies [30,33–35], including index cases predicted in our analysis.

With our approach we could separate likely transmitters from other clustered cases, rather than treating each cluster as a single unit, and so could associate biological, epidemiological, and demographic variables with transmission. Our dataset has 2 major shortcomings—namely the low number of transmission links with enough statistical support and the fact that only 21 clusters met the criteria for the analysis—and thus our clusters are not necessarily representative of the whole population. Still, our data suggest that certain risk and epidemiological factors are enriched among the transmitters, while others are depleted. In addition, we corroborate that individuals with negative sputum smear status can contribute to transmission (40% of index cases), as has been discussed previously [36,37]. Larger population-based datasets including a larger number of clusters meeting the criteria will help to better define the exact role of these factors.

Our selection of TransPhylo as a tool to trace transmission was driven by the necessity of considering potential unsampled cases. There are other similar approaches that do not take unsampled cases into account [38] or that use a model more suited to environmental reservoirs [39,40]. In addition, we could not make predictions for some transmission clusters due to the limited observed within-cluster diversity, as anticipated previously [41]. Thus, our analysis focused on those events that we could robustly estimate. It is important to note that predictions may be sensitive to molecular rate variations. We focused our discussion on analyses using a molecular rate that is appropriate for MTBC lineage 4 strains, which dominate the local setting. However, other settings will need to calibrate the model with a different rate as it is becoming apparent that the rate for different lineages may vary [25].

The fact that we estimate that approximately 35% of transmission events occurred before symptom onset could have several explanations. Patient-reported times of symptom onset are subjective, and if symptoms were mild, disease may not have been recognized for some time. However, in most cases the time difference between symptom onset and transmission spans several weeks or even months. Recently it has been speculated that sub-clinical transmission may exist and be facilitated by unrelated cough [42]. Here we show evidence for transmission during the asymptomatic phase of disease, in which the transmission probability is lower than during exacerbated disease, but non-negligible [42,43].

There is evidence from clinical trials of sputum smear positive individuals who are otherwise healthy being potential transmitters [3]. This is in line with recent evidence showing a spectrum of different disease states (from almost healthy to diseased [4]) and the possibility that a percentage of those traditionally considered latently infected TB cases in reality are active TB cases with sub-clinical disease [3,44]. Our transmission analysis suggests that sub-clinical disease may jeopardize current TB control strategies, in line with results from epidemiological models [44].

A limitation of our method is that we could not test it on other publicly available genomic datasets. One reason is because it is difficult to obtain cases associated epidemiological data, especially those related to symptom onset (which is a key variable of our study). Despite this, we validated our method by (1) conducting sensitivity analyses using different TransPhylo parameters and (2) comparing the predicted transmission time for foreign-born TB cases with the time of immigration. Nevertheless, the lack of published datasets with the relevant epidemiological data highlights the need to incorporate these variables in prospective TB epidemiological studies.

In conclusion, our individual-based transmission inference method demonstrates that many likely transmitters, including index cases, are missed by contact investigations. Strikingly, a substantial proportion of these transmitters likely spread TB during sub-clinical disease. Future work aligning biomarkers and epidemiological research will help to elucidate host biomarkers of transmission during the spectrum of TB infection, to design better TB control strategies.

## Supporting information

S1 Fig Histograms of model parameters. (PDF)

S2 Fig Trace plot of model parameters coloured by the simulated clock rates. (PDF)

S3 Fig Genetic network reconstruction of all transmission clusters used in the study (part 1). (PDF)

S4 Fig Genetic network reconstruction of all transmission clusters used in the study (part 2). (PDF)

S5 Fig Weighted mean number of unsampled TB cases under different simulated clock rates. (PDF)

S6 Fig Resampled median time of first transmissions (part 1). (PDF)

S7 Fig Resampled median time of first transmissions (part 2). (PDF)

S8 Fig Resampled median time of first transmissions (part 3). (PDF)

S9 Fig Resampled median time of first transmissions (part 4). (PDF)

S10 Fig Resampled median time of first transmissions (part 5). (PDF)

S11 Fig Resampled median time of first transmissions (part 6). (PDF)

S12 Fig Resampled median time of first transmissions (part 7). (PDF)

S13 Fig Density of time to diagnosis among those cases estimated to have caused more than 1 versus 0–1 secondary cases. (PDF)

S1 Table Meta-analysis table for different MTBC clock rates published. (PDF)

S2 Table Characteristics and genetic information about selected clusters. (PDF)

S3 Table Comparison table between the clustered cases detected in the global ongoing study and those included in this research. (PDF)

S4 Table Comparison between time of arrival of foreign nationals in the region and probability of transmitting TB in the region before symptoms. (PDF)

S1 Text Additional methods. (PDF)

S2 Text Additional results. (PDF)

## Abbreviations

MCMC: Markov chain Monte Carlo
MJN: median joining network
MLAG: most likely ancestral genotype
MTBC: *Mycobacterium tuberculosis* complex
OR: odds ratio
TB: tuberculosis
WGS: whole genome sequencing
