# Supplementary material for: High-resolution mapping of tuberculosis transmission: Whole genome sequencing and phylogenetic modelling of a cohort from Valencia Region, Spain
Source: PLoS Med. 2019 Oct 31;16(10):e1002961. doi: 10.1371/journal.pmed.1002961 (PMC6822721; doi:10.1371/journal.pmed.1002961)

S1 Fig. Histograms of model parameters *neg* --- within-host diversity, *off.r* --- first parameter of negative binomial offspring distribution, or equivalently the basic reproduction number, and *pi* --- sampling rate, from the posterior. *tp1* and *tp2* --- independent *TransPhylo* runs for the first and second timed tree simulated from the simulator function *simulateOutbreak*; *tpj* --- *TransPhylo* run on the two-timed trees with parameter sharing. Credible intervals are shown in blue and the true parameter values in red. The credible intervals cover 95% quantile of the posteriors.

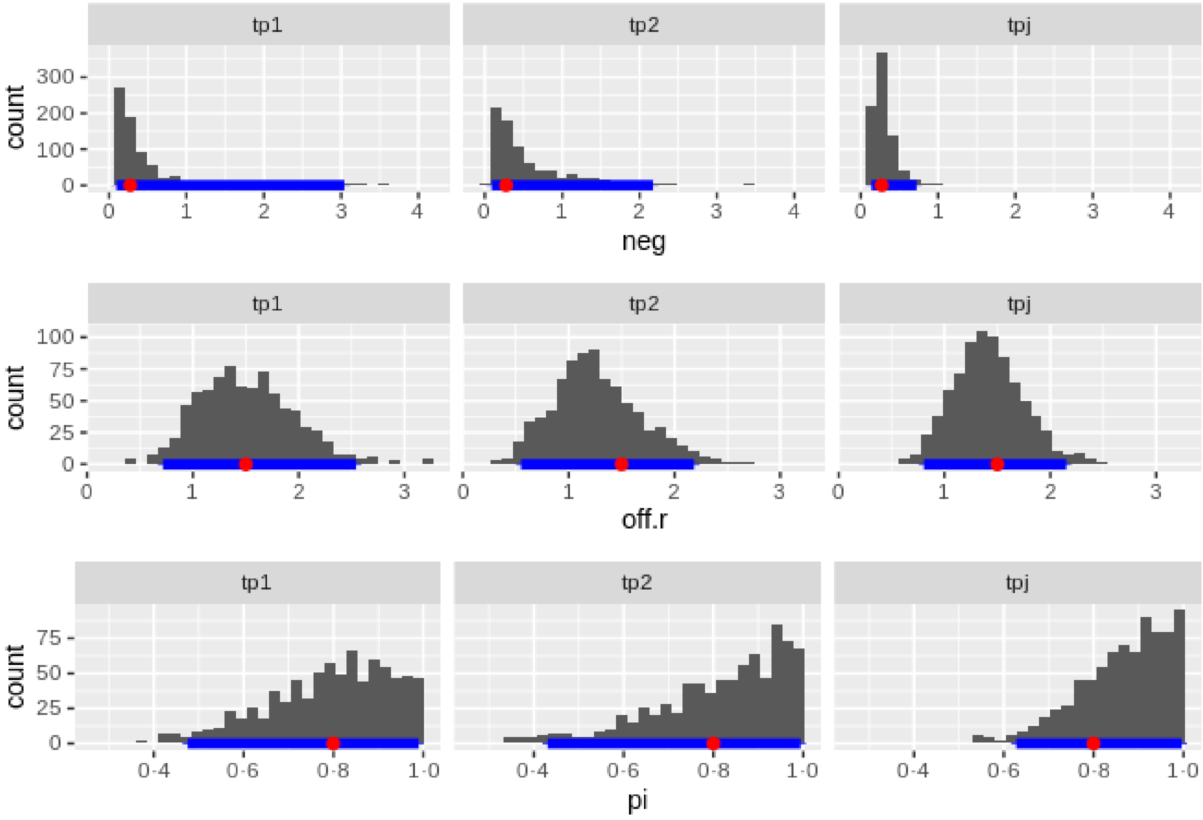

Supplement: S1 Fig — (PDF) [file pmed.1002961.s001.pdf]
