# Supplementary material for: High-resolution mapping of tuberculosis transmission: Whole genome sequencing and phylogenetic modelling of a cohort from Valencia Region, Spain
Source: PLoS Med. 2019 Oct 31;16(10):e1002961. doi: 10.1371/journal.pmed.1002961 (PMC6822721; doi:10.1371/journal.pmed.1002961)

S2 Fig. Trace plot of model parameters colored by the simulated clock rates. The figure shows the trace plot of within-host diversity (*neg*), the offspring distribution parameter (*off.r*) and the sampling proportion (*pi*) from the MCMC run.

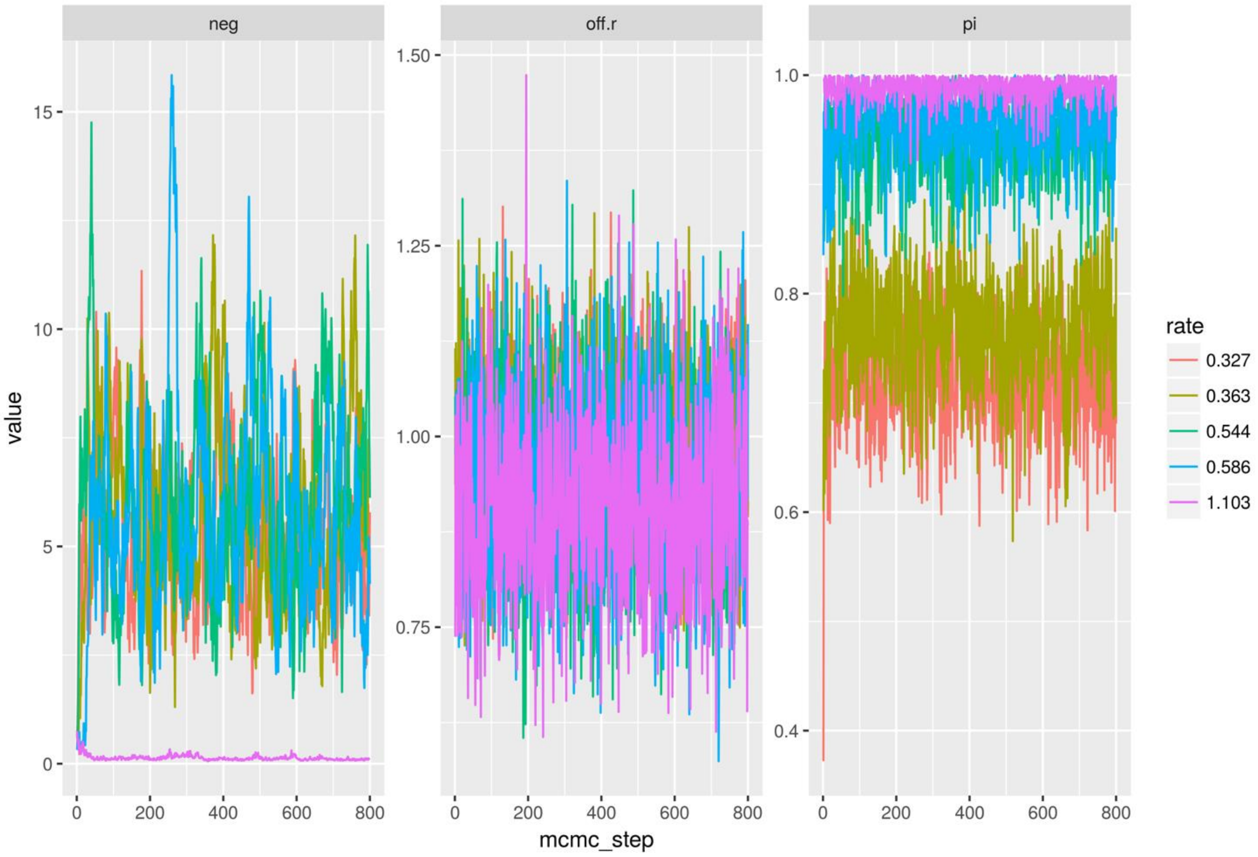

Supplement: S2 Fig — (PDF) [file pmed.1002961.s002.pdf]
