# Supplementary material for: High-resolution mapping of tuberculosis transmission: Whole genome sequencing and phylogenetic modelling of a cohort from Valencia Region, Spain
Source: PLoS Med. 2019 Oct 31;16(10):e1002961. doi: 10.1371/journal.pmed.1002961 (PMC6822721; doi:10.1371/journal.pmed.1002961)

**S3 Fig. Genetic Network reconstruction of all transmission clusters used in the study (*part 1*).** The first diagnosed case is colour coded in green while the rest are colour coded blue. The Most Likely Index Case (brown colour) is the sample that has the same genotype as the predicted Most Likely Ancestral Genotype (red colour). The number inside brackets represents SNP difference between each isolate. The arrow denotes the high likely direction of the transmission.

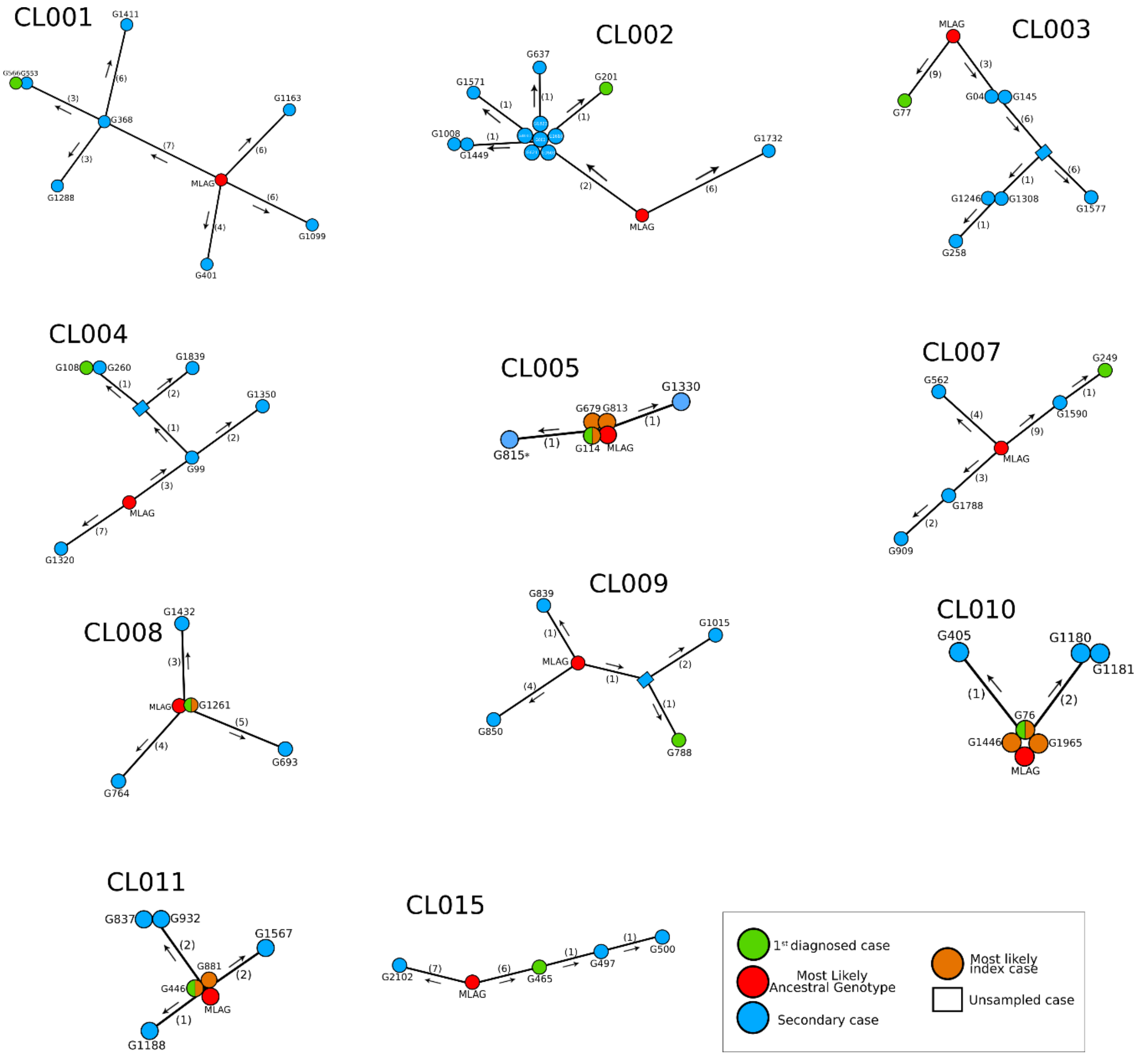

Supplement: S3 Fig — (PDF) [file pmed.1002961.s003.pdf]
