# Supplementary figures and images for: High-resolution mapping of tuberculosis transmission: Whole genome sequencing and phylogenetic modelling of a cohort from Valencia Region, Spain
Source: PLoS Med. 2019 Oct 31;16(10):e1002961. doi: 10.1371/journal.pmed.1002961 (PMC6822721; doi:10.1371/journal.pmed.1002961)

**S5 Fig. Weighted mean number of unsampled cases under different simulated clock rates.**

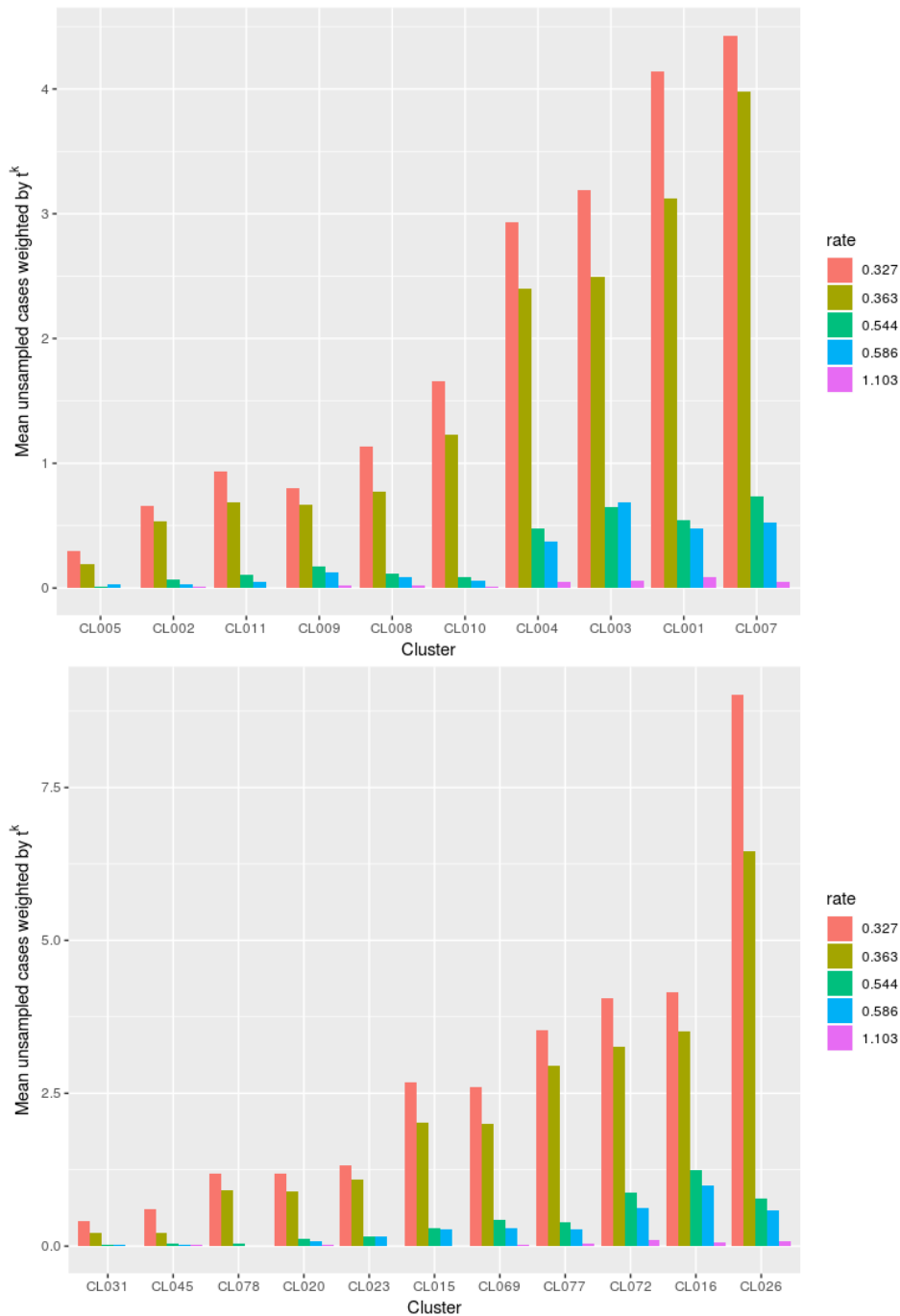

Supplement: S5 Fig — (PDF) [file pmed.1002961.s005.pdf]
