# Supplementary material for: High-resolution mapping of tuberculosis transmission: Whole genome sequencing and phylogenetic modelling of a cohort from Valencia Region, Spain
Source: PLoS Med. 2019 Oct 31;16(10):e1002961. doi: 10.1371/journal.pmed.1002961 (PMC6822721; doi:10.1371/journal.pmed.1002961)

S6 Fig. **Resampled median time of first transmissions.** The graph represents the median time of the first highly likely transmission for cases where the posterior probability of transmitting is greater than 0.5, under a clock rate of 0.363. For each case, the diagnosis time (square), and, where known, the symptom onset time (triangle) are added. Lighter colours indicate higher transmission probabilities. The range of the error bar indicates the 0.25 and 0.75 quantile.

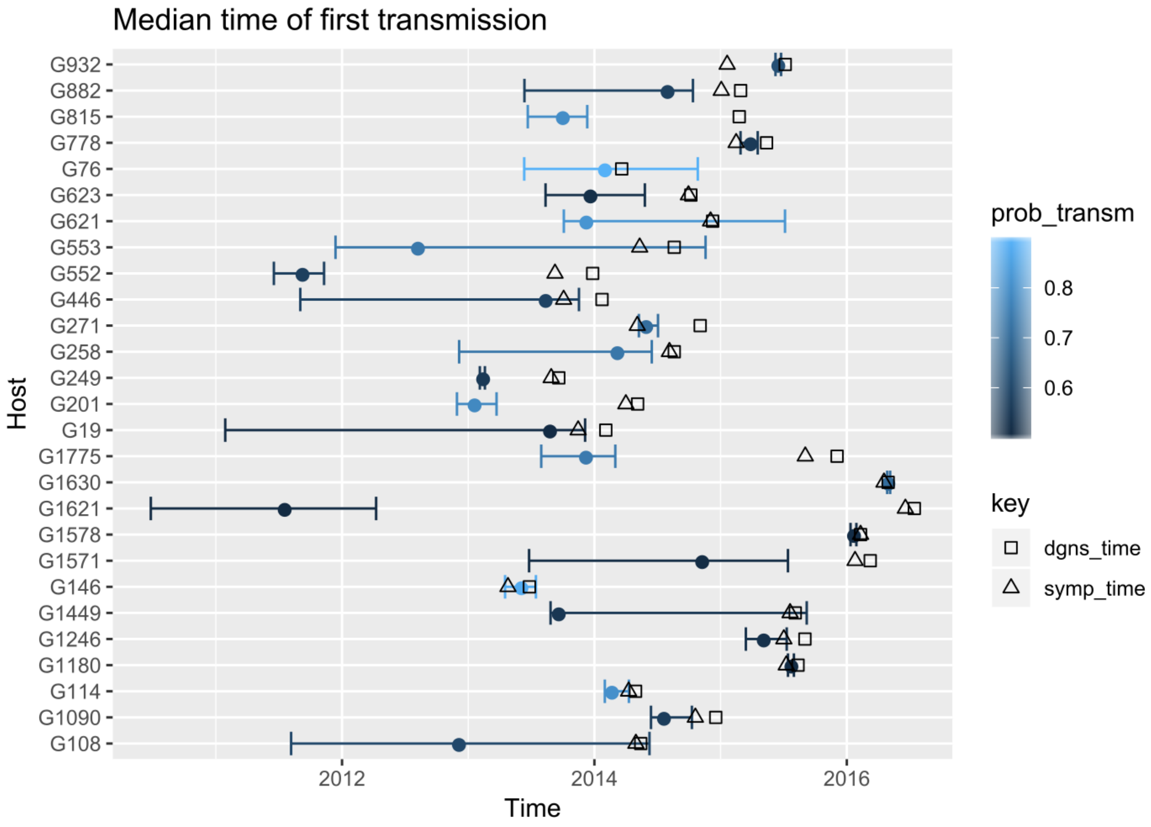

Supplement: S6 Fig — (PDF) [file pmed.1002961.s006.pdf]
