# Supplementary material for: High-resolution mapping of tuberculosis transmission: Whole genome sequencing and phylogenetic modelling of a cohort from Valencia Region, Spain
Source: PLoS Med. 2019 Oct 31;16(10):e1002961. doi: 10.1371/journal.pmed.1002961 (PMC6822721; doi:10.1371/journal.pmed.1002961)

S12 Fig. Resampled median time of first transmissions. The graph represents the median time of the first highly likely transmission for all cases, corresponding to a posterior probability of transmitting of ≥0.2, under a clock rate of 0.544. For each case, the diagnosis time (square), and, where known, the symptom onset time (triangle) are added. Lighter colours indicate higher transmission probabilities. The range of the error bar indicates the 0.25 and 0.75 quantile.

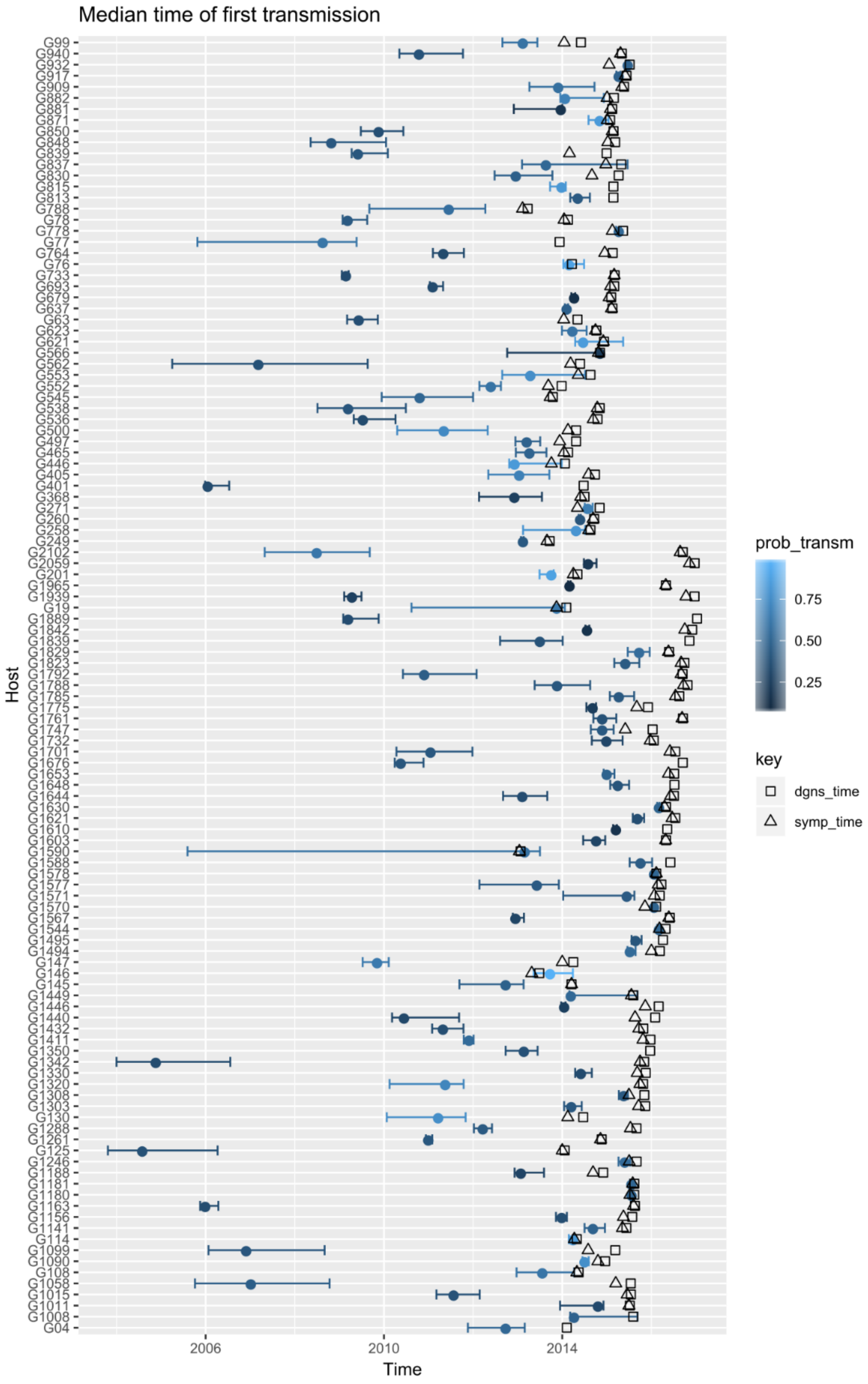

Supplement: S12 Fig — (PDF) [file pmed.1002961.s012.pdf]
