# Supplementary material for: High-resolution mapping of tuberculosis transmission: Whole genome sequencing and phylogenetic modelling of a cohort from Valencia Region, Spain
Source: PLoS Med. 2019 Oct 31;16(10):e1002961. doi: 10.1371/journal.pmed.1002961 (PMC6822721; doi:10.1371/journal.pmed.1002961)

**S13 Fig. Density of times to diagnosis among those cases estimated to have caused more than one vs 0-1 secondary cases.** Estimates were derived under a clock rate of 0.363 and are collected over all posterior transmission events. The means time since the infection of the transmitter to active disease of the secondary case (thus including latency periods) are 4.88 years (those infecting more than 1 secondary case) vs 3 years (those infecting 0 or 1 secondary cases).

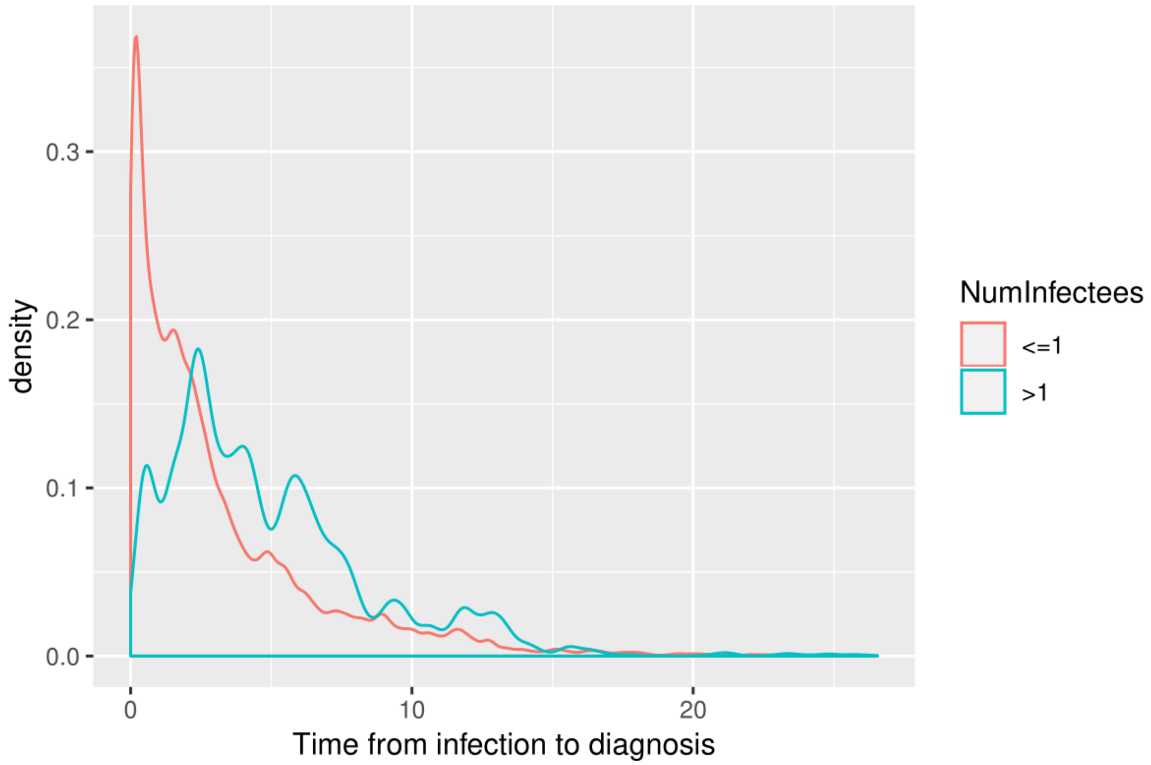

Supplement: S13 Fig — (PDF) [file pmed.1002961.s013.pdf]
