# Supplementary material for: High-resolution mapping of tuberculosis transmission: Whole genome sequencing and phylogenetic modelling of a cohort from Valencia Region, Spain
Source: PLoS Med. 2019 Oct 31;16(10):e1002961. doi: 10.1371/journal.pmed.1002961 (PMC6822721; doi:10.1371/journal.pmed.1002961)
Supplement: S1 Table — (PDF) [file pmed.1002961.s014.pdf]

S1 Table. Meta-analysis table for different published MTBC clock rates.

Reference	Publication Year	MTBC Lineage	No. of samples	Clock rate (genome per year)
Ford et al.[5]	2011	L4	33	0.34
Walker et al.[6]	2013	All	390	0.50
Bryant et al[7]	2013	L1, L2, L3 and L4	199	0.30
Ford et al.[8]	2013	L4	36	0.36
Roetzer at al.[2]	2013	L4	86	0.44
Bos et al.[9]	2014	All	261	0.22
Merker et al. [10]	2015	L2	110	0.44
Luo et al.[11]	2015	L2	393	0.20
Eldhom et al.[12]	2015	L4	252	0.29
Kay et al.[13]	2015	L4	165	0.22
Duchêne et al.[1]	2016	All and L4	261 and 252	0.24 and 0.25
Bjorn-Mortensen et al.[14]	2016	L4	182	0.47
Liu et al.[15]	2018	All	160	0.20
Merker et al.[16]	2018	L2	220	0.41
Duchêne et al.[17]	2018	L2	110	0.41
Rutaihwa et al.[18]	2018	L2	308	0.59
Brynildsrud et al.[19]	2018	L4	269	0.21
Meehan et al.[20]	2018	L4 and L5	324	0.14
