# Supplementary material for: High-resolution mapping of tuberculosis transmission: Whole genome sequencing and phylogenetic modelling of a cohort from Valencia Region, Spain
Source: PLoS Med. 2019 Oct 31;16(10):e1002961. doi: 10.1371/journal.pmed.1002961 (PMC6822721; doi:10.1371/journal.pmed.1002961)
Supplement: S2 Table — (PDF) [file pmed.1002961.s015.pdf]

S2 Table. Characteristics and genetic information about selected clusters. The table shows characteristics of the clusters including numbers of isolates and SNPs.

Cluster ID	Number of isolates	Number of unique strains	Alignment length (SNP)
CL001	8	1	36
CL002	12	7	12
CL003	7	2	26
CL004	6	1	16
CL005	5	2	2
CL007	5	0	19
CL008	4	0	12
CL009	4	0	9
CL010	6	3	4
CL011	6	2	5
CL015	4	1	14
CL016	6	3	16
CL020	4	0	13
CL023	4	0	15
CL026	7	1	18
CL031	5	1	5
CL045	4	1	2
CL069	5	1	14
CL072	5	1	19
CL077	6	1	31
CL078	4	1	3
