# Supplementary material for: High-resolution mapping of tuberculosis transmission: Whole genome sequencing and phylogenetic modelling of a cohort from Valencia Region, Spain
Source: PLoS Med. 2019 Oct 31;16(10):e1002961. doi: 10.1371/journal.pmed.1002961 (PMC6822721; doi:10.1371/journal.pmed.1002961)
Supplement: S3 Table — (PDF) [file pmed.1002961.s016.pdf]

S3 Table. Comparison table between the clustered cases detected in the global ongoing study and those selected in this research.

Characteristic	Global clustered cases* (*n*=325)	Selected clustered cases‡ (n=109)
**Age (years)**		
< 18	23 (7%)	11 (10%)
19-34	78 (24%)	20 (18%)
35-65	194 (59.7%)	66 (61%)
> 65	35 (10.7%)	12 (11%)
**Gender**		
Female	114 (35%)	33 (30%)
Male	211 (64.9%)	76 (70%)
**Place of birth**		
Spanish-born	230 (70.7%)	80 (73%)
Foreign-born	95 (29.2%)	29 (27%)
**Sputum smear**		
Positive	197 (60.6%)	66 (61%)
Negative	126 (38.8%)	41 (38%)
**Disease type**		
Pulmonary	290 (89.2%)	100 (92%)
Extrapulmonary	35 (10.7%)	9 (8%)
**Alcoholism**	69 (21.2%)	25 (23%)
**Diabetes**	34 (10.4%)	13 (12%)
**HIV infected**	24 (7.3%)	10 (9%)
**Social exclusion**	36 (11%)	13 (12%)
**Health care workers**	9 (2.7%)	5 (5%)
**Imprisioment**	22 (6.7%)	8 (7%)
**Diagnostic delay (≥100 days)**	61 (18.7%)	23 (21%)
**≤30 days**	125 (38.4%)	46 (42%)
**31-60 days**	77 (23.7%)	25 (23%)
**61-89 days**	35 (10.8%)	14 (13%)
**≥90 days**	76 (23.4%)	32 (29%)
**Contact tracing transmission**	77 (23.7%)	24 (22%)

* Number of clustered cases detected in the ongoing population study (n=785). These samples involves a total of 121 transmission clusters.

‡ Number of clustered cases selected for this study. These samples involves a total of 21 transmission clusters.
