# Supplementary material for: High-resolution mapping of tuberculosis transmission: Whole genome sequencing and phylogenetic modelling of a cohort from Valencia Region, Spain
Source: PLoS Med. 2019 Oct 31;16(10):e1002961. doi: 10.1371/journal.pmed.1002961 (PMC6822721; doi:10.1371/journal.pmed.1002961)
Supplement: S4 Table — (PDF) [file pmed.1002961.s017.pdf]

S4 Table. Comparison between time of arrival of foreign nationals in cluster and probability of transmitting TB in the region before symptoms.

Case ID	Number of cluster	Probability	Transmitter	Tr_lower	Estimated Transmission year	Tr_upper	Year of arrival	Transmission since arrival (years)	Transmission	Symptoms time
G1630	CL072	0.70	YES	2016.32	2016.33	2016.35	2015	1.33	after	2016.30
G201	CL002	0.77	YES	2012.81	2012.99	2013.19	2000	12.99	after	2014.25
G146	CL045	0.90	YES	2013.29	2013.42	2013.66	2008	5.42	after	2013.32
G1761	CL045	0.12	NO	2013.39	2013.43	2013.46	2015	-1.57	before	2016.69
G1099	CL001	0.15	NO	2002.79	2002.91	2003.06	2003	-0.09	before	2014.58
G1939	CL077	0.16	NO	2005.23	2005.33	2005.60	2009	-3.67	before	2016.78
G368	CL001	0.28	NO	2011.13	2012.03	2013.15	2014	-1.97	before	2014.41
G940	CL020	0.31	NO	2008.58	2008.92	2009.93	2015	-6.08	before	2015.31
