# Supplementary material for: High-resolution mapping of tuberculosis transmission: Whole genome sequencing and phylogenetic modelling of a cohort from Valencia Region, Spain
Source: PLoS Med. 2019 Oct 31;16(10):e1002961. doi: 10.1371/journal.pmed.1002961 (PMC6822721; doi:10.1371/journal.pmed.1002961)
Supplement: S1 Text — (PDF) [file pmed.1002961.s018.pdf]

High-resolution mapping of tuberculosis transmission: whole genome sequencing and phylogenetic modelling of a cohort from Valencia Region, Spain

S1 Text

Supplementary Methods

Ongoing population study

The present study is part of an ongoing population TB study. This global study consisted in the recollection and WGS analysis of a total of 785 positive TB clinical samples during 2014-2016. Using SNPs distances between isolates (≤15 SNPs), we detected that 41% (n=325) of all samples were in transmission. Although the majority of clustered cases comprised two samples, we detected transmission clusters that involved up to 12 TB cases. From this first analysis, we obtained the samples that we used in the present study. We selected to 21 genomic clusters (17.3% of all clusters identified) that corresponded 117 isolates (36% of the total transmission). These genomic groups that had at least four cases and had 2 SNPs difference between all samples involved. Furthermore, we made a comparison analysis to see whether our sampling selection was representative of the whole population (**S3 Table**).

Timed tree reconstruction

Although the accepted value for the TB substitution rate in the community is approximately 0·3-0·5 substitutions per genome per year[1,2], our data seem to suggest that the rate may vary both between clusters and within clusters. For example, in some clusters the SNP distances between pairs of hosts are not consistent with the case timings. For example, a host sampled earlier in time can seem to have accumulated more SNPs than a host sampled later (compared to inference of an ancestral sequence for a cluster), or vice versa. In such cases, an estimated timed phylogenetic tree using a low clock rate (as is normally assumed in TB) would place the earliest sequence in the cluster quite far back in time compared to the most recent sampled case.

We therefore need to incorporate rate uncertainty in the inference framework. However, one challenge is that we do not know if and how rates vary across clusters, and furthermore, although *treedater*[3] allows us to fit a relaxed clock, the consequence of increased number of parameters and lack of signal contained in small cluster data mean that the branch length estimates may not be reliable. Therefore, we adopt a simple approach whereby instead of using a single timed phylogenetic tree for each cluster, we sample clock rates from a known distribution and use treedater to estimate timed trees for all clusters by fixing the clock rate to be in the range of one of our sampled rate values with a margin of ±δ. So for each sampled clock rate, we obtain timed tress corresponding to all clusters and we used *TransPhylo* [4] and the method outlined below to infer the transmission trees. We then pool the transmission trees for each clock rate. By inspecting this combined posterior, we can compare between rates and see if any of the interesting quantities are sensitive to changes in clock rate. We perform a meta-analysis of 18 publications reporting clock rates per year from different studies of MTBC (see **S1 Table**). We obtained a mean rate of 0.32 (±0.022-0.44) but with very wide range of values (0.14-0.59). Thus, we chose to use a log-normal distribution with log-scale mean and standard deviation of -0.7 and 0.5, respectively, for the sampling distribution of the clock rate and $\delta = 0.2$.

Transmission inference

We develop our method of simultaneous transmission inference on many clusters based on *TransPhylo*, a Bayesian method to reconstruct transmission trees from pathogen phylogeny. In *TransPhylo*, an MCMC method is used to draw samples from the posterior distribution of transmission trees and model parameters given a timed phylogenetic tree reconstructed from sequenced isolates (1).

$$\mathbb{P}(T,\theta|P) \propto \mathbb{P}(P|T,\theta)\mathbb{P}(T|\theta)\mathbb{P}(\theta), \quad (1)$$

where T is transmission tree, P is timed tree and θ collects the model parameters. The transmission tree is represented by a matrix whose columns are the times of infection, times of sampling and the infectors, and whose rows correspond to infected individuals. If a case is not sampled, then the corresponding entry for time of sampling is empty. In the posterior trees that *TransPhylo* produces, the number of rows in T can be variable across iterations, because of the addition/removal of unsampled cases; reversible-jump MCMC is used in *TransPhylo* to account for changes of dimensionality.

The transmission tree contains information about who infected whom and when, and also whether a case is sampled or not. The timed phylogenetic tree shows the ancestral history of a set of pathogen isolates sampled from hosts and is constructed using known methods of phylogenetic tree reconstruction. The *TransPhylo* posterior thus reflects our updated belief of the transmission pattern and epidemiological parameters after observing the timed tree of the sequences.

In practice, especially in low-incidence settings, we often define transmission clusters based on our knowledge of the genomics and the epidemiology of cases, such that we are quite confident that transmissions occurred within clusters and were less likely between clusters. This makes it more amenable to analyze multiple clusters simultaneously than to work with a single large phylogeny of all sequences, because these clusters tend to be separated by long branches and a method like *TransPhylo* will need to place many unsampled cases along these branches and so will not explore transmission within clusters efficiently.

In order to develop a framework for simultaneous transmission inference, a straightforward extension to (1) is to carry out our Bayesian inference in an augmented tree and parameter space. More precisely, let $\boldsymbol{T}$, $\boldsymbol{P}$ and $\boldsymbol{\theta}$ be elements in the respective joint space of n clusters, that is, $\boldsymbol{T} = (T_1,\ldots.T_n)$ with T_i the transmission tree for cluster i, and similarly for $\boldsymbol{P}$ and $\boldsymbol{\theta}$, then

$$\mathbb{P}(\mathbf{T},\Theta|\mathbf{P}) \propto \prod_{i=1}^{n}\mathbb{P}(P_i|T_i,\theta_i)\mathbb{P}(T_i|\theta_i)\mathbb{P}(\theta_i), \quad (2)$$

assuming independence between clusters. MCMC simulation of (2) proceeds as it would in (1), with each step consisting of separately updating parameters and trees for all clusters. This would be no different from independently running *TransPhylo* once for each cluster. In order to allow information to be shared between clusters, we decompose θ into shared and non-shared parts, $\theta = (\theta^s, \theta^{ns})$. The posterior distribution becomes

$$\mathbb{P}(\mathbf{T}, \Theta|\mathbf{P}) \propto \prod_{i=1}^{n} \mathbb{P}(P_i|T_i, \theta_i^{ns}, \theta^s)\mathbb{P}(T_i|\theta_i^{ns}, \theta^s)\mathbb{P}(\theta_i^{ns})\mathbb{P}(\theta^s). \tag{3}$$

Note that θ^s does not have index *i* because it is the same for all clusters. The update of θ^s is based on the Metropolis-Hastings ratio of likelihoods of all clusters.

With the above framework, not only can we handle the statistical inference with multiple transmission clusters simultaneously, we can also choose which parameters should be shared. The latter has both epidemiological and computational implications; if we believe that certain parameters, such as the basic reproduction number (the expected number of secondary infections from any primary infection) and/or the sampling rate are similar across clusters, then we can easily encode this belief into (3). This offers great computational savings as the number of parameters is significantly reduced — avoiding $(n - 1)\mathrm{n}(\theta^s)$ parameter estimations where $\mathrm{n}(\theta^s)$ denotes the number of parameters in θ^s.

Supplementary References

1. Duchêne S, Holt KE, Weill F-X, Le Hello S, Hawkey J, Edwards DJ, et al. Genome-scale rates of evolutionary change in bacteria. Microb Genomics. 2016;2.

2. Roetzer A, Diel R, Kohl TA, Rückert C, Nübel U, Blom J, et al. Whole Genome Sequencing versus Traditional Genotyping for Investigation of a Mycobacterium tuberculosis Outbreak: A Longitudinal Molecular Epidemiological Study. PLOS Med. 2013;10: 1–12. doi:10.1371/journal.pmed.1001387

3. Volz EM, Frost SDW. Scalable relaxed clock phylogenetic dating. Virus Evol. 2017;3: vex025. doi:10.1093/ve/vex025

4. Didelot X, Fraser C, Gardy J, Colijn C. Genomic infectious disease epidemiology in partially sampled and ongoing outbreaks. Mol Biol Evol. 2017;34: 997–1007.

5. Ford CB, Lin PL, Chase MR, Shah RR, Iartchouk O, Galagan J, et al. Use of whole genome sequencing to estimate the mutation rate of Mycobacterium tuberculosis during latent infection. Nat Genet. 2011;43: 482–486. doi:10.1038/ng.811

6. Walker TM, Ip CL, Harrell RH, Evans JT, Kapatai G, Dedicoat MJ, et al. Whole-genome sequencing to delineate Mycobacterium tuberculosis outbreaks: a retrospective observational study. Lancet Infect Dis. 2013;13: 137–146. doi:10.1016/S1473-3099(12)70277-3

7. Bryant JM, Schürch AC, van Deutekom H, Harris SR, de Beer JL, de Jager V, et al. Inferring patient to patient transmission of Mycobacterium tuberculosis from whole genome sequencing data. BMC Infect Dis. 2013;13: 110. doi:10.1186/1471-2334-13-110

8. Ford CB, Shah RR, Maeda MK, Gagneux S, Murray MB, Cohen T, et al. Mycobacterium tuberculosis mutation rate estimates from different lineages predict substantial differences in the emergence of drug-resistant tuberculosis. Nat Genet. 2013;45: 784–790. doi:10.1038/ng.2656

9. Bos KI, Harkins KM, Herbig A, Coscolla M, Weber N, Comas I, et al. Pre-Columbian mycobacterial genomes reveal seals as a source of New World human tuberculosis. Nature. 2014;514: 494–497. doi:10.1038/nature13591

10. Merker M, Blin C, Mona S, Duforet-Frebourg N, Lecher S, Willery E, et al. Evolutionary history and global spread of the Mycobacterium tuberculosis Beijing lineage. Nat Genet. 2015;47: 242–249. doi:10.1038/ng.3195

11. Luo T, Comas I, Luo D, Lu B, Wu J, Wei L, et al. Southern East Asian origin and coexpansion of *Mycobacterium tuberculosis* Beijing family with Han Chinese. Proc Natl Acad Sci. 2015;112: 8136–8141. doi:10.1073/pnas.1424063112

12. Eldholm V, Monteserin J, Rieux A, Lopez B, Sobkowiak B, Ritacco V, et al. Four decades of transmission of a multidrug-resistant Mycobacterium tuberculosis outbreak strain. Nat Commun. 2015;6: 7119. doi:10.1038/ncomms8119

13. Kay GL, Sergeant MJ, Zhou Z, Chan JZ-M, Millard A, Quick J, et al. Eighteenth-century genomes show that mixed infections were common at time of peak tuberculosis in Europe. Nat Commun. 2015;6: 6717. doi:10.1038/ncomms7717

14. Bjorn-Mortensen K, Soborg B, Koch A, Ladefoged K, Merker M, Lillebaek T, et al. Tracing Mycobacterium tuberculosis transmission by whole genome sequencing in a high incidence setting: a retrospective population-based study in East Greenland. Sci Rep. 2016;6: 33180. doi:10.1038/srep33180

15. Liu Q, Ma A, Wei L, Pang Y, Wu B, Luo T, et al. China's tuberculosis epidemic stems from historical expansion of four strains of Mycobacterium tuberculosis. Nat Ecol Evol. 2018;2: 1982–1992. doi:10.1038/s41559-018-0680-6

16. Merker M, Barbier M, Cox H, Rasigade J-P, Feuerriegel S, Kohl TA, et al. Compensatory evolution drives multidrug- resistant tuberculosis in Central Asia. Evol Biol. : 31.

17. Duchene S, Duchene DA, Geoghegan JL, Dyson ZA, Hawkey J, Holt KE. Inferring demographic parameters in bacterial genomic data using Bayesian and hybrid phylogenetic methods. BMC Evol Biol. 2018;18: 95. doi:10.1186/s12862-018-1210-5

18. Rutaihwa LK, Menardo F, Stucki D, Gygli SM, Ley SD, Malla B, et al. Multiple Introductions of Mycobacterium tuberculosis Lineage 2–Beijing Into Africa Over Centuries. Front Ecol Evol. 2019;7: 112. doi:10.3389/fevo.2019.00112

19. Brynildsrud OB, Pepperell CS, Suffys P, Grandjean L, Monteserin J, Debech N, et al. Global expansion of *Mycobacterium tuberculosis* lineage 4 shaped by colonial migration and local adaptation. Sci Adv. 2018;4: eaat5869. doi:10.1126/sciadv.aat5869

20. Meehan CJ, Moris P, Kohl TA, Pecerska J, Akter S, Merker M, et al. The relationship between transmission time and clustering methods in Mycobacterium tuberculosis epidemiology. EBioMedicine. 2018; doi:https://doi.org/10.1016/j.ebiom.2018.10.013
