# Supplementary material for: High-resolution mapping of tuberculosis transmission: Whole genome sequencing and phylogenetic modelling of a cohort from Valencia Region, Spain
Source: PLoS Med. 2019 Oct 31;16(10):e1002961. doi: 10.1371/journal.pmed.1002961 (PMC6822721; doi:10.1371/journal.pmed.1002961)
Supplement: S2 Text — (PDF) [file pmed.1002961.s019.pdf]

High-resolution mapping of tuberculosis transmission: whole genome sequencing and phylogenetic modelling of a cohort from Valencia Region, Spain

S2 Text

Supplementary Results

Simulations

We simulated two outbreaks using *TransPhylo*'s simulator function *simulateOutbreak*, with different model parameters: 1) *neg* — within-host diversity; 2) *off.r* — the first parameter of the negative binomial offspring distribution, or equivalently the basic reproduction number; and 3) and *pi* — the sampling rate. Because of the stochastic nature of the simulator, we obtain different timed phylogenetic trees. We then apply the new joint inference routine to infer the model parameters and compare them with those obtained from running *TransPhylo* on the clusters separately. For both the two independent runs and the joint routine the same number of MCMC iterations (10^4) was used, with a burn-in of 20%. Hence the computer time of the joint routine is about the same as the total time of running the two independent runs sequentially. **S1 Fig** shows the performance of the shared parameter approach ("tpj" for TransPhylo- Joint) compared to individual *TransPhylo* runs.

TransPhylo parameters

S2 Fig shows the traces of the parameters in the MCMC (downsampled and ignoring a burn-in). For both *neg* and *off.r*, an exponential prior *Exp*(1) was used; while a Beta prior *Beta*(5, 1) was used for *pi*. We see that the offspring distribution parameter, which is also the R_0, is very robust to changes in clock rate. A high sampling proportion of 0.7 was observed even with the lowest clock rate, reflecting our prior belief of high sampling. In addition, the *neg* parameter is not affected by small changes in clock rate, however it is significantly lower if the clock rate is very high relative to the other rates.
